# The complete mitochondrial genome of *Psammomys obesus* (Rodentia: Muridae)

**DOI:** 10.1080/23802359.2017.1422396

**Published:** 2018-01-08

**Authors:** Yanhong Lan, Mengjia Liu, Yi Cao

**Affiliations:** Microbiology and Metabolic Engineering of Key Laboratory of Sichuan Province, College of Life Science, Sichuan University, Chengdu, China

**Keywords:** *Psammomys obesus*, mitochondrial genome, protein-coding genes, phylogeny

## Abstract

The fat sand rats (*Psammomys obesus*) can easily induce obesity and acquire type 2 diabetes mellitus when they are fed with high-carbohydrate diets. *P. obesus* is often used as an animal model for studies on diabetes and obesity. We described 16,592 bp of *P. obesus* mtDNA that contains 13 protein-coding genes (PGCs), two rRNA genes (12S rRNA and 16S rRNA), 22 transfer RNA (tRNA) genes, and one control region (D-loop). The complete mitochondrial genome sequence provided here would be useful for further understanding the evolution of ratite and conservation genetics of *P. obesus*.

The fat sand rats (*Psammomys obesus*) belong to the genus *Psammomys* within the subfamily Gerbillinae of the family *Gerbillinae* that are widely distributed in North Africa and the Middle East, ranging from Mauritania to the Arabian Peninsula. *P. obesus* can easily acquire non-insulin-dependent diabetes mellitus and the complications associated with diabetes are (cataracts, pancreatic atrophy, and impaired renal function) from high-caloric foods(Kaiser et al. 2005, 2012; Ouadda et al. 2009).

Apart from previous studies about some biological characters, causes of population depletion, embryonic development, and population genetic diversity (Kaissling et al. 1975; FichetCalvet et al. 1999; Shenbrot 2004; Ouadda et al. 2009; Hargreaves et al. 2017), molecular studies about *P. obesus* are limited. No mitochondrial genome of *P. obesus* is available until now. We will determine the mitochondrial genome of *P. obesus* in this study. The total genomic DNA was extracted from blood of a male adult *P. Obesus* in Israel county (31°47'N, 35°13'E) and sequenced with Illumina HiSeq 2000 (San Diego, CA) (Hargreaves et al. 2017), the sample of *P. Obesus* was stored in NCBI (Accession no. SAMN06061930). The complete mitogenome was assembled with MIRA 4.0.2 (San Francisco, CA) (Burlibasa et al. 1999) and MITObim 1.9 (Hahn et al. 2013). The mitochodrial genome was annotated and drawn by MitoFish 3.30 (http://mitofish.aori.u-tokyo.ac.jp/) (Figure 1).

**Figure 1.**
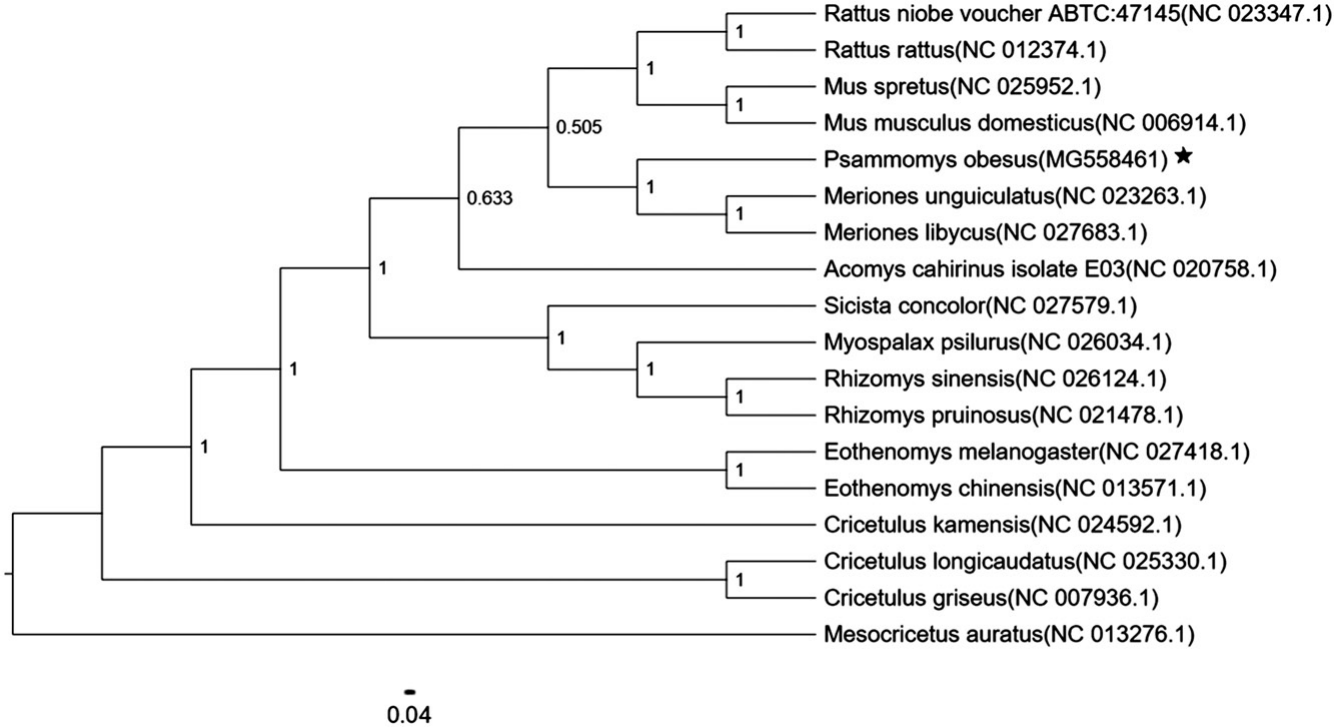
Neighbor-joining molecular phylogenetic tree of 18 species of Glires based on complete mitogenome sequences, with *M. auratus* as an outgroup. The asterisk indicates the individual sampled in this study. GenBank accession numbers are indicated in brackets.

The complete mitochondrial genome of *P. obesus* is a double-stranded, circular DNA 16,592 bp in total length (GenBank accession no. MG558461), and includes 13 protein-coding genes, two ribosomal RNA genes (12S rRNA and 16S rRNA), 22 tRNA genes, and one control region (D-loop). The contents of A, G, T, and C are 33.09%, 24.01%, 29.63%, and 12.67%, respectively. GC contents of mitochondrial genome are 36.68%. Twelve of the PCGs use complete (TAA) or incomplete (T-) stop codon. The 12S rRNA and 16S rRNA genes are 951 and 1575 bp, respectively. The lengths of 22 tRNA genes are from 60 bp (tRNA-Ser) to 75 bp (tRNA-Leu). The D-loop length is 1163 bp and lies between the tRNA-Pro and tRNA-Phe genes.

The phylogenetic analysis of 18 mitochondrial genomes using MEGA 7 (Kumar et al. 2016) in which *Mesocricetus auratus* is used as the outgroup indicated that *P. obesus* and *Meriones. unguiculatus* are the most closely related species (Figure 1). The mitogenome of *P. obesus* would contribute to the understanding of the phylogeny and evolution of Rodentia.
